# Early biochemical and radiographic response after one cycle of [^177^Lu]Lu-PSMA I&T radioligand therapy in metastatic castration-resistant prostate cancer patients

**DOI:** 10.1007/s00259-023-06326-w

**Published:** 2023-07-21

**Authors:** Wojciech Cytawa, Robin Hendel, Bartłomiej Tomasik, Franz-Xaver Weinzierl, Thorsten Bley, Jacek Jassem, Andreas Schirbel, Andreas K. Buck, Ralph A. Bundschuh, Philipp E. Hartrampf, Rudolf A. Werner, Constantin Lapa

**Affiliations:** 1 Department of Nuclear Medicine, University Hospital Würzburg, Würzburg, Germany; 2 Department of Nuclear Medicine, Medical University of Gdańsk, Gdańsk, Poland; 3 Department of Radiology, University Hospital Würzburg, Würzburg, Germany; 4 Department of Oncology and Radiotherapy, Faculty of Medicine, Medical University of Gdańsk, Gdańsk, Poland; 5 Nuclear Medicine, Faculty of Medicine, University of Augsburg, Stenglinstr. 2, 86156 Augsburg, Germany

**Keywords:** Metastatic castration-resistant prostate cancer, [^177^Lu]Lu-PSMA I&T, Radioligand therapy, Early response assessment

## Abstract

**Purpose:**

The aim of this study was to investigate very early radiographic PSMA PET response after one cycle of [^177^Lu]Lu-PSMA I&T radioligand therapy (RLT) of metastatic castration-resistant prostate cancer (mCRPC) and to assess its role in predicting overall response and survival.

**Methods:**

This retrospective study enrolled 40 mCRPC patients who were treated with a median of 3 (2–9) [^177^Lu]Lu-PSMA I&T RLT cycles. Biochemical response was based on the relative change of serum PSA according to PCWG3 criteria, while radiographic response referred to the relative change of PSMA-derived total viable tumor volumes expressed as total lesion PSMA (TLP).

**Results:**

After one cycle of RLT, biochemical partial response (PR) was seen in 8/40 (20.0%), stable disease (SD) in 22/40 (55.0%), and progressive disease (PD) in 10/40 (25%) patients. In PSMA PET, very early molecular PR was observed in 12 (30.0%), SD in 19 (47.5%), and PD in 9 (22.5%) subjects. The PSA and TLP nadir were achieved after a median of 1 (1–5) and 2 (1–6) cycles, respectively. Nineteen (47.5%) patients showed overall biochemical PR, 11 (27.5%) had SD, and 10 (25%) experienced PD. In PSMA-directed PET, 4 patients experienced molecular complete response (CR), 24 (60.0%) had PR, 4 (10.0%) SD, and 8 (20.0%) PD. Early biochemical or radiographic response was not associated with longer overall survival (OS). Overall biochemical responders had a nearly significantly longer median OS (22.7 months) than non-responders (14.4 months, *p* = 0.08). Early PSA progression was associated with shorter OS (12.2 months), compared to biochemical SD/PR (18.7 months, *p* = 0.09).

**Conclusion:**

In this retrospective cohort, there was no association between early PSMA PET radiographic response and overall survival; hence, treatment should not be prematurely discontinued. In contrast, early PSA progression after one cycle of [^177^Lu]Lu-PSMA I&T RLT was an indicator of overall progression and poor clinical outcome.

**Supplementary information:**

The online version contains supplementary material available at 10.1007/s00259-023-06326-w.

## Introduction

Prostate cancer (PCa) is the second most common malignancy in men worldwide (first in developed countries) and a leading cause of cancer-related deaths [1, 2]. More than half of the patients initially treated with curative-intent experience relapse within several years after completion of therapy [3]. Moreover, about 12% of individuals present with metastases at the time of initial diagnosis, with numbers increasing up to ~ 20% in cohorts with high-risk disease [4]. Thus, the majority of patients usually require implementation of androgen deprivation therapy (ADT), the most common first-line systemic treatment, during the course of disease. However, continuous ADT eventually leads to castration-resistance and the patients experience further disease progression. Metastatic castration-resistant prostate cancer (mCRPC) has various treatment options, including taxane-based chemotherapy (docetaxel and cabazitaxel), novel androgen axis drugs (abiraterone, enzalutamide, apalutamide, darolutamide), bone-seeking agents ([^223^Ra]Ra-dichloride), or poly-ADP-ribose polymerase (PARP) inhibitors for patients with mutations in DNA repair genes (rucaparib and olaparib) [5]. Alternatively, radioligand therapy (RLT) targeting the prostate-specific membrane antigen (PSMA)—a transmembrane glycoprotein highly expressed on prostate cancer cells—can be offered. Lutetium-177 labeled PSMA ligands already proved effective in retrospective studies [6, 7] and prospective clinical trials [8, 9], which recently led to the approval of [^177^Lu]Lu-PSMA-617 under the trade name Pluvicto® by the European Medicines Agency. Appropriate selection of candidates for PSMA RLT requires, among others, imaging with PSMA-directed positron emission tomography/computed tomography (PET/CT), using gallium-68 or fluorine-18-labeled radioligands in order to confirm overexpression of PSMA on tumor lesions—a pre-requisite for therapy [10].

The other benefit of performing PSMA PET before consecutive cycles of RLT is the ability of monitoring treatment response. This, however, is still a subject of scientific debate and the criteria how to assess a treatment response remain to be defined. One option is to use PERCIST (PET response criteria in solid tumors) *per analogiam* with [^18^F]FDG PET/CT [11]; however, this concept suffers from similar limitations as RECIST (response evaluation criteria in solid tumors), being limited only to the measurement of single-target lesions. Assessment of the PSMA-derived total tumor volume before and after therapy in [^68^Ga]Ga-PSMA-11 PET/CT was first proposed by Schmuck et al. [12] and Schmidkonz et al. proved its efficiency in response assessment of PCa [13], similarly to other malignancies, mainly lymphomas, in [^18^F]FDG PET [14, 15]. Recent RECIP (response evaluation criteria in PSMA-imaging) criteria [16] also take into consideration the presence or absence of new PSMA-positive lesions.

An extension of the PSMA-positive tumor volume method is based on the estimation of the total lesion PSMA (TLP) expression, which should reflect the total tumor burden, additionally taking into account the uptake intensity of individual lesions. This concept originates from the fact that PSMA accumulation in the tumor is directly associated with the number of tumor cells, which has been investigated in preclinical studies [17].

Biochemical response defined by PCWG3 (Prostate Cancer Working Group 3) as ≥ 50% decrease of baseline PSA serum level [18] occurs in 30–65% of patients undergoing [^177^Lu]Lu-PSMA RLT [19], although, in some cases, it may only appear after several cycles of therapy [20]. Recently, early molecular response assessment after two cycles of RLT has been presented [21]. The authors showed that the change in TLP outperforms conventional PSA-based response assessment and predicts overall survival in mCRPC. However, very early changes in tumor PSMA expression after one cycle of RLT have not been studied so far.

In this study, we retrospectively investigated the value of TLP obtained from PSMA PET/CT for treatment response assessment to [^177^Lu]Lu-PSMA I&T RLT after the first treatment cycle.

## Material and methods

### Compliance with ethical standards

Between June 2015 and August 2020, 81 consecutive patients with mCRPC were treated with [^177^Lu]Lu-PSMA I&T RLT on a compassionate use basis in compliance with the German Pharmaceutical Act §13 (2b) and in accordance with the Declaration of Helsinki, paragraph 37 for “Unproven Interventions in Clinical Practice.” The study protocol was approved by the local ethics committee (no. 20220502 01). The decision to use PSMA RLT in every patient was taken by the institutional interdisciplinary tumor board. All patients gave written informed consent to undergo RLT with subsequent follow-up.

### Patient recruitment

The following inclusion criteria, consistent with the guidelines of German Society of Nuclear Medicine [22] and the VISION trial criteria [9], were considered: histopathological confirmation of PCa, presence of unresectable (or not amenable to curative local treatment) metastases, castration resistant disease, other on-label options of treatment of mCRPC completed or contraindicated, progression of disease by PSA level and imaging, PSMA-positive lesions confirmed in pre-therapeutic PSMA PET/CT imaging, white blood cell count (WBC) > 3000/μL, platelet count > 75,000/μL, creatinine < twofold the upper limit of normal (ULN), AST and ALT < fivefold ULN, and no myelosuppressive therapy within 6 weeks prior to RLT.

Out of the initial 81 patients, 63 received at least 2 cycles of RLT, of whom 40 had the first follow-up [^68^Ga]Ga-PSMA I&T PSMA PET/CT after the first cycle of treatment and were finally included in the study. After the launch of this novel PSMA RLT in 2015 at our hospital, we were particularly vigilant about the status of the PSMA biotarget in our patients and recommended a control PSMA PET scan before each administration of [^177^Lu]Lu-PSMA I&T. We assumed that the very early identification of patients who would not benefit from RLT would allow us to stop an ineffective treatment and consider other options. We came to the conclusion that the additional financial costs and radiation burden would be justified in this context.

Main patients’ characteristics, including previous therapies prior to RLT, staging data, and basic laboratory parameters are summarized in Table 1. Clinical outcome data were retrieved from the hospital medical records. The cohort has been partially described in [23–28].

**Table 1:** Baseline characteristics of the 40 patients treated with [^177^Lu]Lu-PSMA I&T

Patient characteristic	Value
Age at the onset of RLT [years]	71.7 ± 8.6 (54–91)
Baseline PSA (ng/mL)	166 (0.1–5000)
Baseline TLP (SUV × mL)	3786.3 (27.6–20,032.2)
Prior radical prostatectomy, *n* (%)	22 (55.0)
Prior primary radical radiotherapy, *n* (%)	6 (15.0)
Prior systemic therapy lines, *n* (%)	
≤ 2	25 (62.5)
≥ 3	15 (37.5)
Metastatic sites, *n* (%)	
Lymph nodes, overall	31 (77.5)
Lymph nodes only	4 (10.0)
Bone, overall	36 (90.0)
Bone only	9 (22.5)
Lymph nodes and bone	27 (67.5)
Visceral	5 (12.5)
Cumulative activity of [^177^Lu]Lu-PSMA I&T (GBq)	18.9 (11.7–54.8)
Hemoglobin (g/dL, normal range: 13.5–16.9)	11.6 ± 1.7 (7.9–14.9)
C-reactive protein (mg/dL, normal range: 0–0.5)	0.3 (0.02–10.0)
Lactate dehydrogenase (U/L, normal range: < 250)	233.5 (118–509)
Alkaline phosphatase (U/L, normal range: 40–130)	99.5 (38–1499)

*RLT*, radioligand therapy. Ranges or percentages are indicated in parentheses in the right column

### Radioligand therapy

RLT cycles were repeated at median intervals of ~ 9 weeks, and added up to 9 cycles depending on response to treatment. Prior to each cycle every patient, in addition to clinical evaluation, had a panel of blood tests including blood count, PSA serum level, electrolytes, and kidney and liver function. Each treatment cycle was performed during a 3-day stay at the radionuclide therapy ward of the Department of Nuclear Medicine at the University Hospital of Würzburg, Germany. [^177^Lu]Lu-PSMA I&T was administered by slow i.v. infusion lasting about 20 min. A median activity of 6.0 (range, 3.0–8.0) GBq of [^177^Lu]Lu-PSMA I&T was used per cycle.

Any adverse events for bone marrow toxicity and tubular extraction rate were graded by National Cancer Institute’s CTCAE (Common Terminology Criteria for Adverse Events) criteria, version 5.0 (2017).

### Preparation of [^177^Lu]Lu-PSMA I&T

A solution of 150 µg PSMA I&T (Scintomics GmbH, Fürstenfeldbruck, Germany) and 7 mg gentisic acid in 600 μL sodium acetate buffer (pH = 4–5) was added to a solution of 6 GBq [^177^Lu]LuCl_3_ in 200 µL of 0.04 M hydrochloric acid (ITG, Garching, Germany) and heated for 35 min at 95 °C. The product was diluted with saline and passed through a sterile filter (0.22 μm) into a sterile syringe. Radiochemical purity was determined being > 98% by reversed phase high-performance liquid chromatography and instant thin-layer chromatography [23].

### PSMA PET/CT imaging

[^68^Ga]Ga-PSMA I&T PET/CT imaging was performed according to a previously described method [29]. Briefly, images were obtained ~ 1 h after i.v. injection of [^68^Ga]Ga-PSMA I&T (mean activity 2 MBq/kg of body weight) on a 64-detector PET/CT scanner (Siemens Biograph mCT 64, Siemens Healthineers AG, Erlangen, Germany). The preparation of [^68^Ga]Ga-PSMA I&T radioligand has been described in detail previously [29]. A monophasic full-dose CT scan was performed after i.v. injection of an iodine-based contrast agent (1 mL/kg body weight of Imeron® 350) for anatomic correlation and attenuation correction.

### Assessment of therapy response

#### PSA response

According to PCWG3 criteria [18], biochemical complete response (CR) was defined as a decrease of serum PSA level to 0 ng/mL, partial response (PR) as a decrease of ≥ 50%, progressive disease (PD) as a rise of ≥ 25%, while stable disease (SD) as a change between – 50% and + 25% of initial PSA. Hence, patients with complete or partial biochemical response were classified as “PSA responders,” while with biochemical progression or stable disease as “PSA non-responders.” Early PSA response was checked after the first cycle of RLT (week 8), while overall (best) PSA response was estimated at the PSA nadir.

#### Radiographic (molecular) response

Each PSMA PET scan was analyzed with a semi-automatic tumor segmentation algorithm using Syngo.Via (version VB40B, Siemens Healthcare GmBH, Erlangen, Germany, 2009–2020). The SUV threshold of ≥ 4 was chosen for segmentation, as this value better separated tumor lesions from areas of high physiological tracer uptake than the threshold of ≥ 3 previously used [21, 30]. After manual exclusion of physiologic uptake sites, such as salivary glands, liver, spleen, kidneys, intestine, ureters, and urinary bladder, a total lesion PSMA (TLP) was calculated from the total tumor volume multiplied by uptake (SUV_mean_) and expressed in SUV × mL. For delineation of metastases localized in organs with high physiologic PSMA expression, e.g., liver, visual assessment and manual segmentation of the lesions were performed.

In analogy to modified PET response criteria in solid tumors (mPERCIST) [11], PSMA PET response assessment was based on the percentage changes of the total lesion PSMA, as previously established by Rosar et al. [21]. The disappearance of all PSMA-avid lesions, resulting in a 100% reduction in TLP, was defined as CR, a decrease in TLP ≥ 30% as PR, an increase of TLP of ≥ 30% as PD, whereas the change of TLP from – 30 to + 30% as SD. Consequently, patients with complete or partial radiographic response were classified as “TLP responders,” while those with radiographic progression or stable disease as “TLP non-responders.” By analogy to PSA response, early radiographic response was assessed after the first cycle of RLT (week 9), whereas overall (best) radiographic response was assessed at the TLP nadir.

Additionally, radiographic response evaluation based on the percentage change of PSMA-positive tumor volume, as previously established by Grubmüller et al. [31], was assessed.

### Survival analysis

Overall survival (OS) was the primary endpoint used in the survival analysis and was measured from the date of the first RLT cycle to patient death from any cause (complete observation) or the date of the last follow-up information (censored observation). Due to the retrospective nature of the study, the follow-up of patients was not standardized.

### Statistical analysis

The statistical analysis was performed with Statistica 13.1 Software (StatSoft Polska, Copyright 2016). The normal distribution of variables was verified by the Shapiro–Wilk W test. Normally distributed values were described as mean ± standard deviation (SD) together with range, while values without normal distribution were described as median (range). Categorical values were expressed as frequencies and proportions. The changes in individual patients’ PSA and TLP levels, sorted by the extent of change, were illustrated by waterfall plots. The temporal dynamics of PSA and TLP were analyzed by Friedman’s ANOVA and subsequently by Friedman-Nemenyi post-hoc tests. Linear correlation was analyzed by Pearson’s correlation coefficient. Cohen’s kappa (*κ*) was used to verify the interrater agreement between PSA and TLP response. Kaplan–Meier analysis was used to compare survival outcomes in subgroups of patients. Both uni- and multivariable Cox regression analyses were performed for different variables in order to elucidate potential predictors of survival. The optimal cut-off for dichotomized values was defined as the point with the most significant (log-rank test) split and rounded to facilitate clinical interpretation [32]. The names of appropriate statistical tests used in the analysis were given when necessary. For all analyses, *p* value < 0.05 was considered statistically significant.

## Results

### Patients’ general characteristics

The mean age of the 40 mCRPC patients included in this study at the time of the first cycle of PSMA RLT was 71.7 ± 8.6 years. The median time interval from primary diagnosis to PSMA-directed therapy was 7.5 (1.9–30.3) years.

At the beginning of therapy, 90% (36/40) of patients presented with multiple bone metastases (BMs), 77.5% (31/40) with lymph node metastases (LNMs), and 12.5% (5/40) with visceral metastases (VMs, 4 with liver, and 1 with adrenal metastases). More details on patients’ characteristics can be found in Table 1. The median number of cycles per patient was 3 (2–9), with the median cumulative activity of [^177^Lu]Lu-PSMA I&T being 18.9 (11.7–54.8) GBq.

### Early biochemical and radiographic response characteristics

After the first cycle of RLT, the median PSA value dropped from 166 (0.1–5000) to 118.5 (0.07–5000) ng/mL. Biochemical PR was seen in 8/40 (20.0%) patients, SD occurred in 22/40 (55.0%), and PD in 10/40 (25%) individuals. None of the patients presented with early biochemical CR.

At PSMA PET imaging, the median initial TLP slightly dropped from 3786.3 (27.6–20032.2) to 3422.9 (10.0–16601.1) SUV × mL. Molecular PR was observed in 12 (30.0%) patients, SD in 19 (47.5%) individuals, while PD was detected in 9 (22.5%) subjects. Complete molecular response was not reported in any of the patients after one RLT cycle (Table 2).

**Table 2:** Concordance between *early* biochemical (PSA) and radiographic (TLP) response in patients undergoing [^177^Lu]Lu-PSMA I&T RLT (*n* = 40)

		TLP			
		Complete response	Partial response	Stable disease	Progressive disease
PSA	Complete response	0	0	0	0
	Partial response	0	5	3	0
	Stable disease	0	6	13	3
	Progressive disease	0	1	3	6
		*κ* = 0.3574			

*PSA*, prostate-specific antigen; *TLP*, total lesion PSMA (total tumor burden)

### Overall (best) biochemical and radiographic response characteristics

The PSA nadir throughout the entire PSMA RLT was achieved after the median of 2 (1–5) cycles and its median value was 92.9 (0.04–5000) ng/mL. None of the patients achieved biochemical CR; however, in one case the PSA level was almost undetectable (0.04 ng/mL). Nineteen (47.5%) patients showed biochemical PR, 11 (27.5%) had SD, and 10 (25%) subjects experienced PD.

The best molecular response was seen after a median of 2 (1–6) cycles, and the median TLP nadir was 1625.2 (0–16601.1) SUV × mL. Four patients experienced molecular CR, with disappearance of all PSMA-avid tumor lesions (exceeding the SUV threshold of 4.0), 24 (60.0%) patients had PR, 4 (10.0%) SD, and 8 (20.0%) PD, with various numbers of new PSMA-positive foci (Table 3).

**Table 3:** Concordance between best *overall* biochemical (PSA) and radiographic (TLP) response in patients undergoing [^177^Lu]Lu-PSMA I&T RLT (*n* = 40)

		TLP			
		Complete response	Partial response	Stable disease	Progressive disease
PSA	Complete response	0	0	0	0
	Partial response	2	15	2	0
	Stable disease	0	8	1	2
	Progressive disease	2	1	1	6
		*κ* = 0.2941			

*PSA*, prostate-specific antigen; *TLP*, total lesion PSMA (total tumor burden)

In general, the concordance between biochemical and radiographic response was moderate, both for early and overall assessment (*κ* = 0.3574 and 0.2941, respectively), with the greatest degrees being achieved in patients with SD in early response and PR in overall assessment (Tables 2 and 3, respectively).

**Fig. 1:**
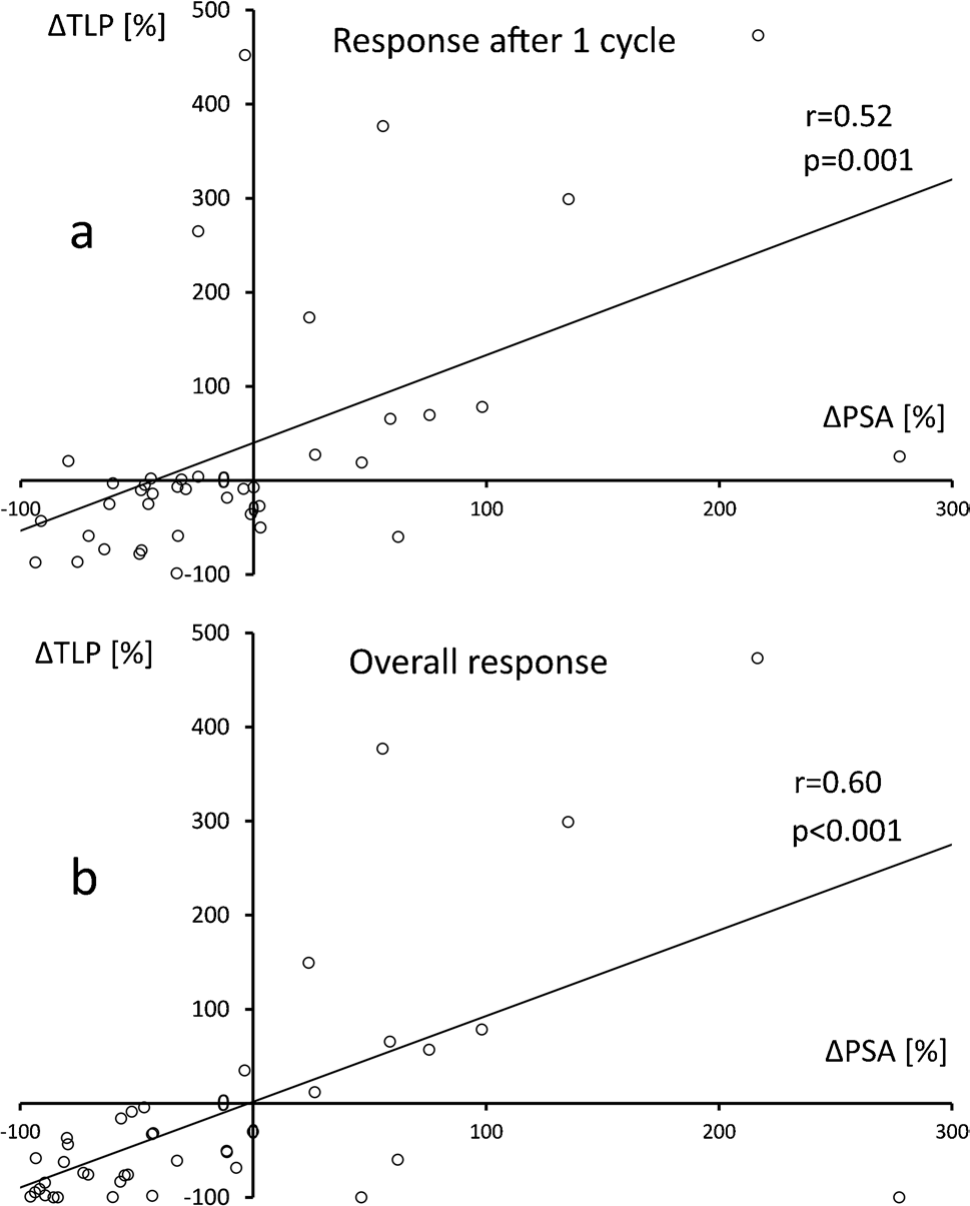
Correlation between early **a** and overall **b** change of TLP (∆TLP [%]) vs. change of PSA (∆PSA [%]) against baseline values after PSMA RLT (*n* = 40). TLP, total lesion PSMA; PSA, prostate-specific antigen

A strong and statistically significant linear correlation was observed between the extent of biochemical and radiographic overall response (*r* = 0.60, *p* < 0.001, Fig. 1b), while moderate correlation was observed for early response (*r* = 0.52, *p* = 0.001, Fig. 1a).

### Early response after one RLT cycle as a predictor of overall response

The median lowest PSA (92.9 ng/mL) achieved in the course of therapy was significantly lower (*p* < 0.001) than the median baseline PSA value (166 ng/mL), while the median PSA after the first cycle of PSMA RLT (118.5 ng/mL) was not significantly lower than the baseline value (*p* = 0.4034).

Similarly, the median TLP nadir (1625.2 SUV × mL) was significantly lower (*p* < 0.001) than the median baseline TLP value (3786.3 SUV × mL), while the median TLP after the first cycle of PSMA RLT (3422.9 SUV × mL) did not significantly differ from the baseline value (*p* = 0.9191).

**Fig. 2:**
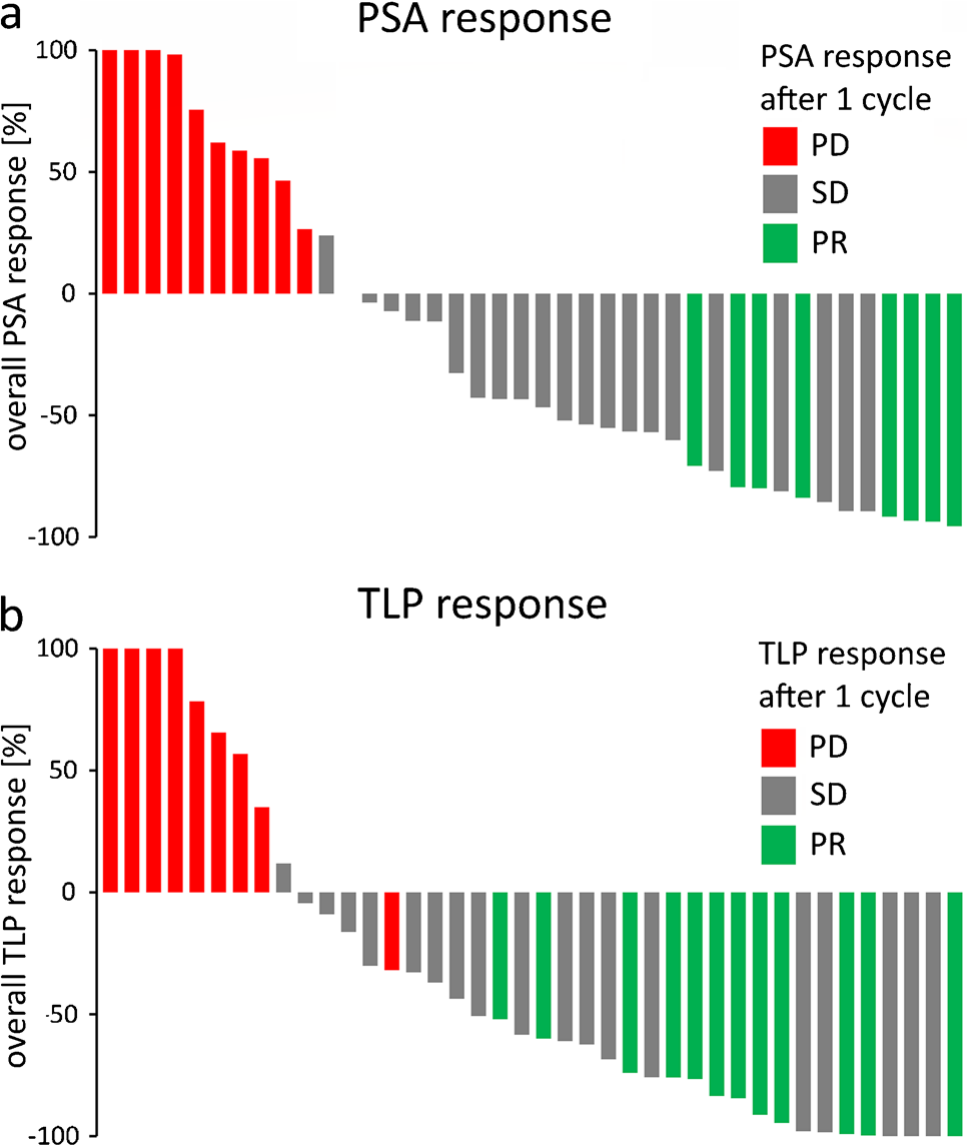
Waterfall plots of 40 patients treated with [^177^Lu]Lu-PSMA I&T showing relative changes in PSA (**A**) and TLP (**B**) in relation to early response characteristics after one cycle of therapy. Values over 100% were cropped for simplicity. PR, partial response; SD, stable disease; PD, progressive disease

The extent of biochemical and molecular response is presented in two waterfall plots (Fig. 2A and 2B, respectively), with the relationship between early and (best) overall responses.

The full concordance between early and overall responses is presented in Tables 4 and 5, for biochemical and radiographic responses, respectively. Eleven out of 22 (50%) patients who had early biochemical SD achieved PR after further treatment (Table 4), while 12/19 (63%) individuals who presented with early radiographic SD had overall PR in PSMA PET imaging (Table 5).

**Table 4:** Concordance between *early* and *overall* biochemical (PSA) response in patients undergoing [^177^Lu]Lu-PSMA I&T RLT (*n* = 40)

		Overall			
		Complete response	Partial response	Stable disease	Progressive disease
Early	Complete response	0	0	0	0
	Partial response	0	8	0	0
	Stable disease	0	11	11	0
	Progressive disease	0	0	0	10
		*κ* = 0.6022			

*PSA*, prostate-specific antigen

**Table 5:** Concordance between *early* and *overall* radiographic (TLP) response in patients undergoing [^177^Lu]Lu-PSMA I&T RLT (*n* = 40)

		Overall			
		Complete response	Partial response	Stable disease	Progressive disease
Early	Complete response	0	0	0	0
	Partial response	1	11	0	0
	Stable disease	3	12	4	0
	Progressive disease	0	1	0	8
		*κ* = 0.4158			

*TLP*, total lesion PSMA (total tumor burden)

### Predictors of survival

The median follow-up time was 17.3 months (95% confidence interval [CI]: 14.3–24.2 months). By the end of the study, 30 (75%) patients had died, all due to progressing mCRPC. No treatment-related deaths were recorded.

**Fig. 3:**
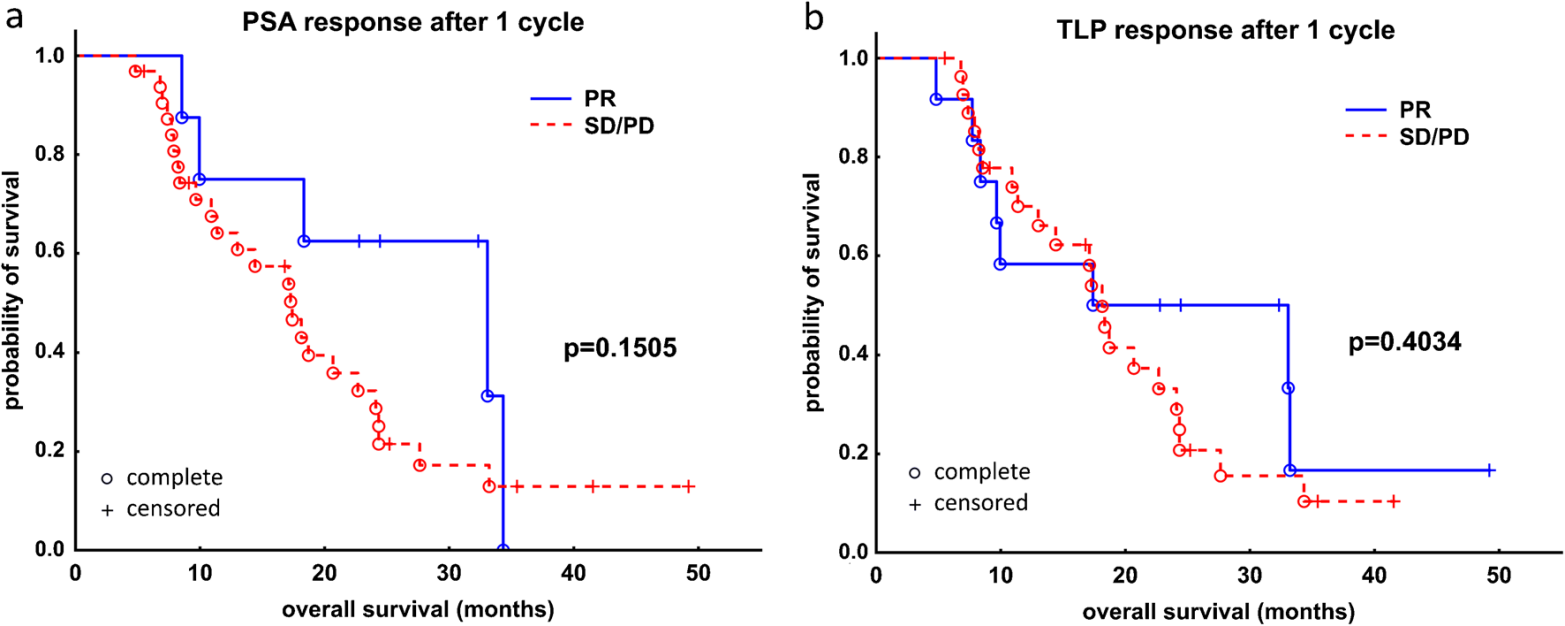
Kaplan–Meier analysis of survival stratified by early biochemical response based on relative change of PSA (**A**) and molecular response based on relative change of TLP (**B**) in 40 patients with mCRPC after one cycle of [^177^Lu]Lu-PSMA I&T. mCRPC, metastatic castration resistant prostate cancer

**Fig. 4:**
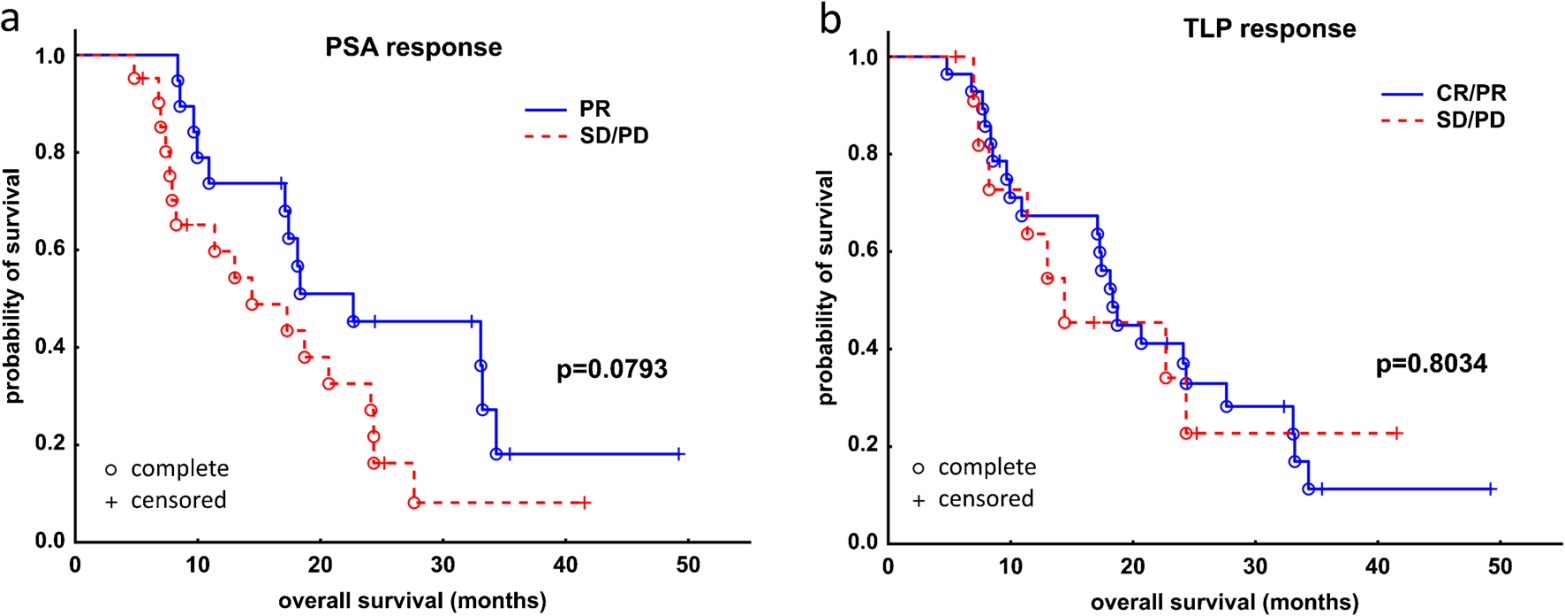
Kaplan–Meier analysis of survival stratified by overall biochemical response based on the relative change of PSA (**A**) and molecular response based on relative change of TLP (**B**) in 40 patients with mCRPC after subsequent cycles of [^177^Lu]Lu-PSMA I&T. mCRPC, metastatic castration resistant prostate cancer

Neither biochemical nor radiographic response after one cycle of RLT was associated with better OS (Fig. 3, panels A and B). Regarding overall response, a trend toward significantly longer survival could be demonstrated in a subgroup of patients who responded to RLT in terms of PSA decrease—biochemical responders had a median OS of 22.7 months (95% CI: 17.1–34.3 months), while non-responders 14.4 months (95% CI: 8.2–24.3 months), *p* = 0.0793 (log-rank test, Fig. 4A). In the radiographic response assessment, the median OS of the responders (18.3 months [95% CI: 17.1–33.3 months]) was insignificantly longer than that of non-responders (14.4 months [95% CI: 11.4–24.3] *p* = 0.8043 (log-rank test, Fig. 4B).

Moreover, early PSA progression was associated with shorter OS, as compared to patients with stable PSA or ≥ 50% PSA decline, with 12.2 (95% CI: 7.4–23.4) vs. 18.7 (95% CI: 17.3–33.2) months, respectively (*p* = 0.0878, Fig. 1SA).

Both uni- and multivariable analyses were performed in order to elucidate potential factors predicting OS among baseline characteristics. The results are summarized in Table 6.

**Table 6:** Univariate and multivariable analysis of potential factors predicting overall survival (OS). P values set in boldface indicate statistical significance. Due to the high PSA and TLP values observed in patients included in the study, the nominal values of these parameters were transformed to express the change in the log hazard per 10 ng/mL and 100 SUV × mL, respectively

	Univariate analysis		Multivariable analysis	
Variable	HR (95%CI)	p value	HR (95%CI)	*p* value
Age (years)	0.98 (0.94–1.03)	0.407		
ECOG performance status				
1 vs. 0	2.27 (0.63–8.15)	0.210		
LNM	0.76 (0.32–1.78)	0.528		
BM	2.29 (0.54–9.67)	0.258		
VM	1.52 (0.58–4.01)	0.399		
Hemoglobin (g/dL)	0.77 (0.59–1.00)	0.051	1.10 (0.80–1.50)	0.571
CRP (mg/dL)	1.30 (1.12–1.51)	** < 0.001**	1.99 (1.27–3.12)	**0.003**
LDH (U/L)	1.01 (1.00–1.01)	**0.001**	1.01 (1.00–1.01)	0.051
Alkaline phosphatase (U/L)	1.00 (1.00–1.00)	**0.018**	1.00 (1.00–1.00)	0.300
Number of prior systemic therapy lines	1.36 (0.87–2.12)	0.172		
Baseline PSA/10 (ng/mL)	1.00 (1.00–1.01)	**0.005**	1.01 (1.00–1.02)	**0.001**
Baseline TLP/100 (SUV × mL)	1.00 (1.00–1.01)	0.116		
PSA response after 1 cycle	0.54 (0.21–1.41)	0.210		
TLP response after 1 cycle	0.72 (0.32–1.62)	0.426		
Overall PSA response	0.52 (0.25–1.09)	0.084	0.35 (0.11–1.01)	0.053
Overall TLP response	0.89 (0.40–2.03)	0.796		
PSA progression after 1 cycle	2.21 (0.98–4.98)	0.055	1.98 (0.70–5.55)	0.196
TLP progression after 1 cycle	0.89 (0.36–2.20)	0.806		

**Fig. 5:**
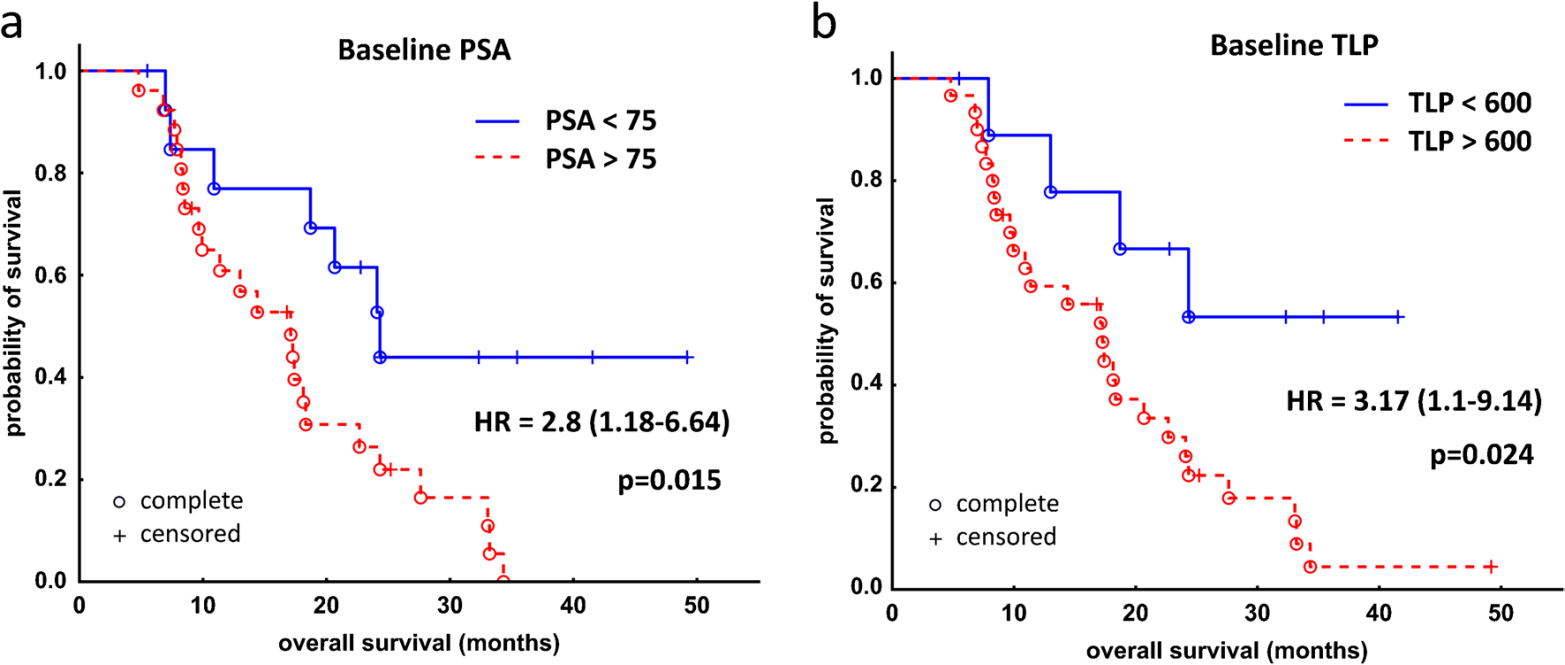
Kaplan–Meier analysis of survival stratified by baseline PSA (**A**) and TLP (**B**) value in 40 patients with mCRPC after subsequent cycles of [^177^Lu]Lu-PSMA I&T. mCRPC, metastatic castration resistant prostate cancer; PSA, prostate-specific antigen (ng/mL); TLP, total lesion PSMA (SUV × mL); HR, hazard ratio

We additionally analyzed PSA and TLP baseline levels to establish the cut-off values allowing dichotomization of patients’ cohorts according to survival prognosis. Baseline PSA was an independent predictor of OS with a hazard ratio (HR) of 2.8 (95% CI: 1.18–6.64), *p* = 0.015 for patients with PSA > 75 ng/mL, relative to patients with PSA < 75 ng/mL (Fig. 5A). When baseline TLP is concerned the parameter achieved statistical significance as an independent factor predicting OS, with a HR of 3.17 (95% CI: 1.1–9.14), *p* = 0.024 for patients with TLP > 600 SUV × mL, relative to patients with TLP < 600 SUV × mL (Fig. 5B).

**Fig. 6:**
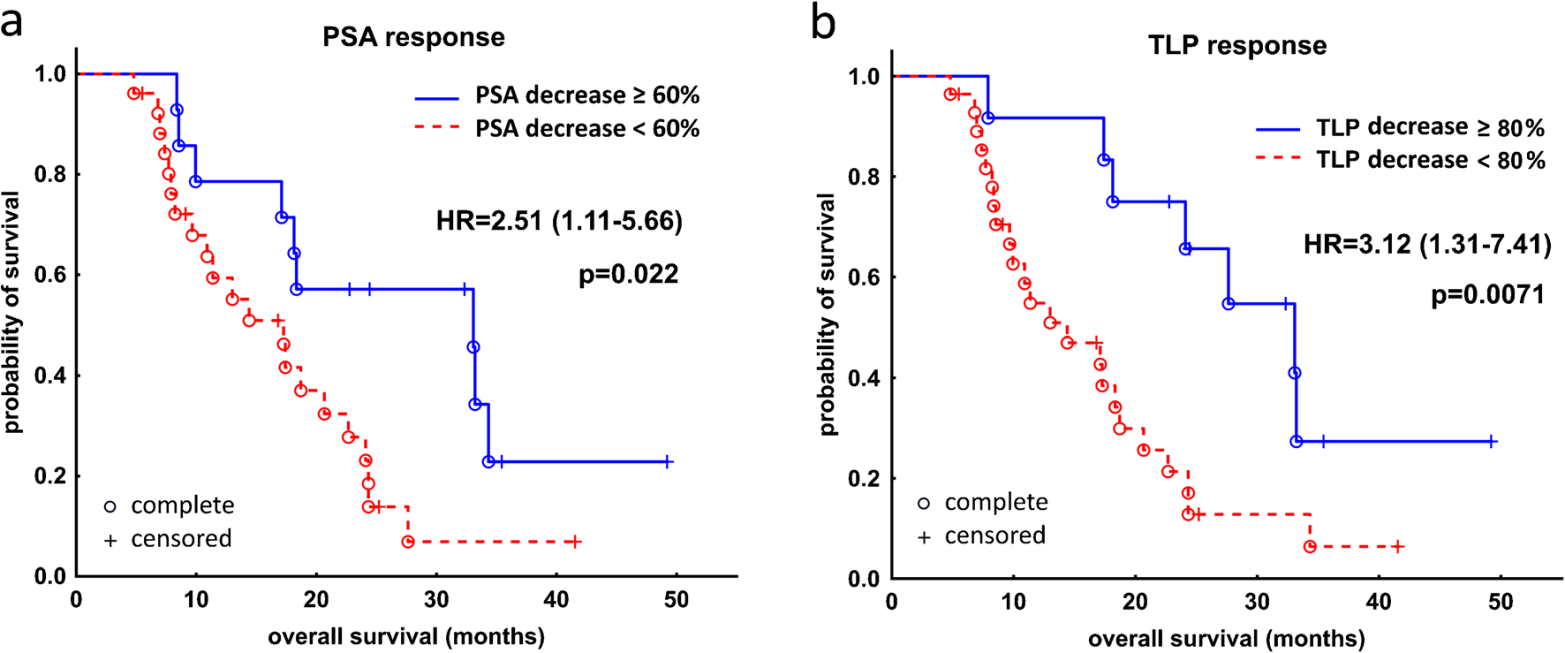
Kaplan–Meier analysis of survival stratified by cut-off percentage change of baseline PSA (**A**) and TLP (**B**) value in 40 patients with mCRPC treated with [^177^Lu]Lu-PSMA I&T. mCRPC, metastatic castration resistant prostate cancer; PSA, prostate-specific antigen; TLP, total lesion PSMA; HR, hazard ratio

Additional dichotomization of cohorts was also performed regarding the percentage change of baseline PSA and TLP. An overall reduction of PSA by ≥ 60% from baseline was associated with better OS with a HR of 2.51 (95% CI: 1.11–5.66, *p* = 0.022) (Fig. 6A). Also, an overall TLP decrease by ≥ 80% from baseline was associated with better OS with a HR of 3.12 (95% CI: 1.31–7.41, *p* = 0.0071) (Fig. 6B). No significant and meaningful dichotomizations could be established for the early response after 1 cycle of PSMA RLT.

## Discussion

In the current study, we retrospectively analyzed the value of very early changes in total lesion PSMA expression (TLP) obtained from PSMA PET/CT imaging for subsequent overall response assessment and OS in mCRPC patients treated with PSMA-directed therapy. We compared potential of this approach with the standard response evaluation based on serum PSA levels, both in early phase after one cycle of PSMA radioligand therapy and after subsequent cycles. Biochemical PR after the first cycle of RLT in our cohort was less frequent (20%), as compared to the response rates of the entire treatment (~ 48%). Early molecular PR was reported in 30% patients, with 70% patients achieving PR or CR after further treatment. The extent of the overall response, expressed both by PSA and TLP change from baseline, was significantly more prominent as compared to the change after the first cycle. Rahbar et al. have also shown that a relevant number of patients (29%) may present with a delayed biochemical response, even if they did not respond to the first cycle of therapy [20]. Here, we prove that the lack of a very early radiographic response to one PSMA RLT cycle (in terms of reduction in total lesion PSMA expression ≥ 30%) is not predictive for overall response and OS, highlighting the cumulative effect of the therapy. In our cohort, exactly half of the patients who presented with early biochemical SD achieved PR during subsequent therapy, while the majority of patients (~ 79%) with early radiographic SD experienced a PR or CR in PSMA PET imaging after further treatment. Importantly, individuals with early PD, either biochemical or radiographic, had only minimal chances of response in further evaluation, with numbers ranging between 0 and ~ 11%, respectively. In terms of biochemical response, the above observation confirms the results of the retrospective study performed by Gafita et al., who analyzed changes in serum PSA levels as early as 6 weeks after initiation of [^177^Lu]Lu-PSMA treatment and concluded that early PSA progression was an indicator of overall progression, with PSA flare-up phenomena being very uncommon [33, 34]. This important clinical finding could help to select a population of patients with PSA progression who could benefit from an early therapy switch. On the other hand, the lack of an early radiographic response at PSMA PET imaging should not prompt an immediate switch in therapy.

The OS of early responders and non-responders (both biochemical and radiographic) in our cohort did not differ. Nearly significantly longer OS was only shown in a subset of patients with overall biochemical response. In overall radiographic response analysis, the survival curves differ temporarily to some extent and there is a trend toward better OS in responders, which could be interpreted as a clinically meaningful benefit. However, the difference between the responders and non-responders did not reach statistical significance, possibly due to the limited number of patients. In an additional evaluation based on the percentage change of PSA and TLP, we found that a reduction in PSA of ≥ 60% from baseline (measured at the PSA nadir) and a reduction in TLP of ≥ 80% were associated with longer OS. Analogous cut-offs could not be established for early response after one cycle of PSMA RLT.

Rosar et al. studied the value of early molecular response after two cycles of [^177^Lu]Lu-PSMA-617 RLT. The authors proved that the change in TLP had a prognostic role for OS and outperformed a PERCIST-based evaluation [21]. The use of TLP parameter mostly addresses the limitations of PERCIST which is based on the comparison of single-target lesions in consecutive studies and gives only a vague idea about the response characteristics of the rest of the tumor burden.

**Fig. 7:**
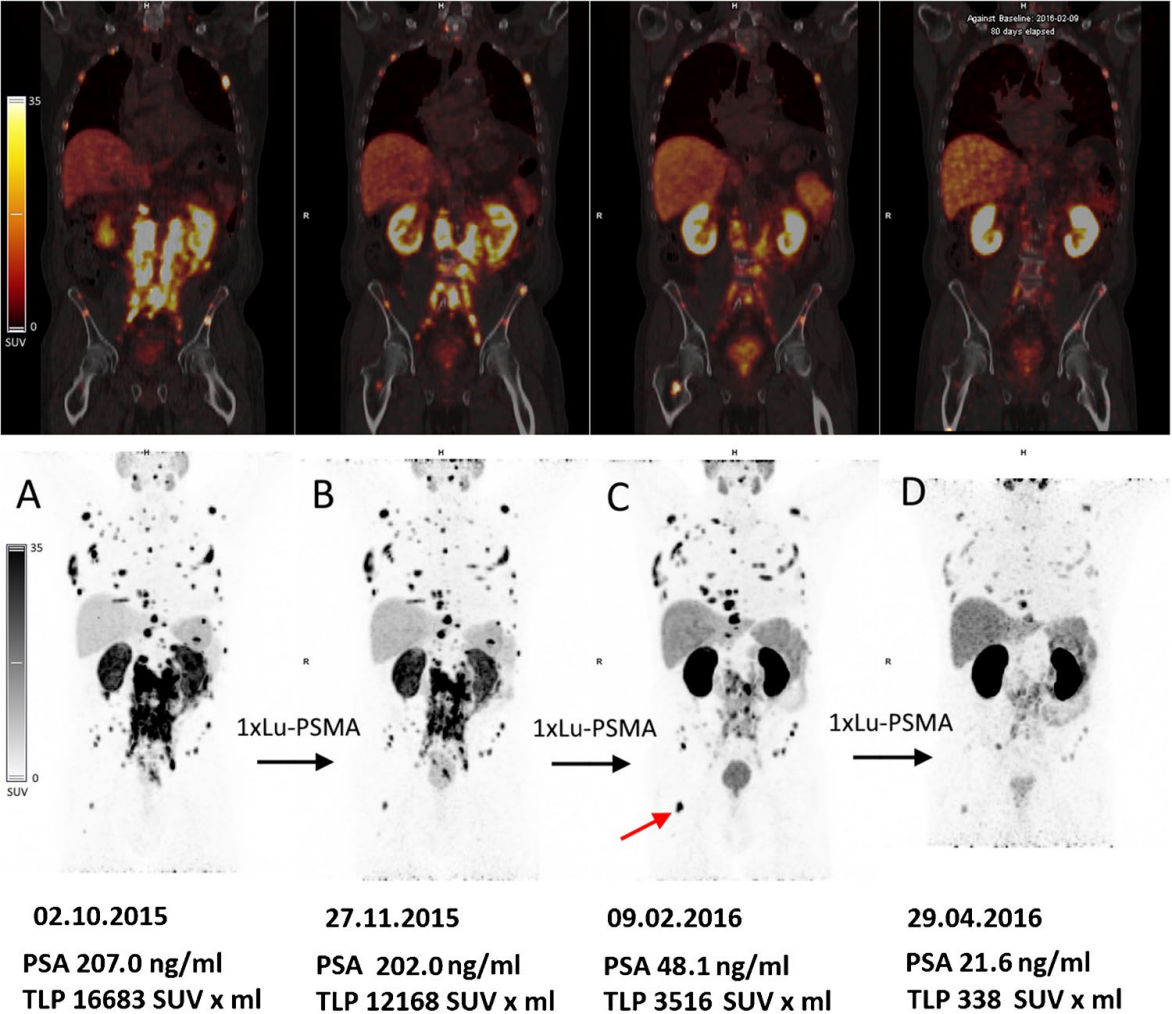
Example of a 61-year-old patient with advanced mCRPC, presenting multiple bone and abdominal lymph node metastases, treated with [^177^Lu]Lu-PSMA I&T. Sub panel **A** represents baseline [^68^Ga]Ga-PSMA PET/CT scan. Sub panel **B** shows [68Ga]Ga-PSMA PET/CT scan after 1 cycle of PSMA RLT. After 3 cycles of PSMA RLT, the patient achieved the best biochemical and radiographic partial response **D**. After the second cycle a transient increase of [^68^Ga]Ga-PSMA uptake was observed in some metastatic lesions, e.g., in the right femur (**C**, red arrow) indicating a “flare effect,” which resolved after the subsequent cycle. The OS from the beginning of RLT was 18 months. mCRPC, metastatic castration resistant prostate cancer

Regarding concordance between biochemical and molecular response, we have observed, similar to other authors [21, 31], certain discrepancies between the two methods. A few patients who responded to RLT biochemically did not present with molecular response. A persistent or even elevated PSMA uptake in tumor foci may be present during the course of therapy, even in patients with a generally good response (example of such case in Fig. 7). It was observed in 9 patients in our cohort (8 with BMs and 1 with LNMs) and could be called a “flare phenomenon.” This term, however, originally refers to an increased uptake of bone-seeking agents or [^18^F]F-fluorodeoxyglucose due to the activation of healing processes after successful therapy of bone metastases [35, 36]. A prolonged PSMA tracer uptake in this context should rather be interpreted as sustaining tumor cells which can survive until a sufficient dose of ionizing radiation is delivered to them. Nevertheless, a flare phenomenon of metastatic PCa has been reported after various forms of treatment, recently also in PSMA PET imaging, after ADT (enzalutamide) [37]. The underlying molecular mechanisms responsible for such intensification of PSMA expression after [^177^Lu]Lu-PSMA RLT are not yet understood and warrant further investigation in preclinical models.

A pseudoprogression may also result from the so-called “tumor sink effect” [38, 39]. It may take place when the majority of lesions in baseline PET respond well to RLT, changing the biodistribution of tracer uptake within the body. Hence, small lesions not visualized before may appear in the follow-up scan due to the greater availability of tracer. It should be pointed out that if the tracer uptake in those lesions exceeds the adopted SUV threshold, it leads to overestimation of the TLP, which may change the category of radiographic response to a less favorable one.

Considering factors predicting survival, two baseline characteristics of our cohort, namely, PSA > 75 ng/mL and TLP > 600 SUV × mL, were associated with worse OS (HR 2.8 and 3.17, respectively). In multivariable analysis two other parameters were also associated with OS, i.e., baseline CRP and LDH (HR 1.99 and 1.01, respectively). These data may help (nuclear medicine) physicians to identify patients who are more likely to benefit from PSMA RLT and achieve better outcomes.

### Study limitations

The main limitations of our study include its retrospective nature and the limited number of patients, which means that the obtained results should be treated with caution.

## Conclusions

In this retrospective cohort, there was no association between early PSMA PET radiographic response and overall survival; hence, treatment should not be prematurely discontinued. In contrast, early PSA progression after one cycle of [^177^Lu]Lu-PSMA I&T RLT was an indicator of overall progression and poor clinical outcome.

### Supplementary information

Below is the link to the electronic supplementary material.

Supplementary file1 (DOCX 606 kb)

Supplementary file2 (XLSX 22 kb)

## Data Availability

The datasets generated during and/or analyzed during the current study are available from the corresponding author on reasonable request.
