# Supplementary material for: Early biochemical and radiographic response after one cycle of [177Lu]Lu-PSMA I&T radioligand therapy in metastatic castration-resistant prostate cancer patients
Source: Eur J Nucl Med Mol Imaging. 2023 Jul 21;50(12):3765–76. doi: 10.1007/s00259-023-06326-w (PMC10547638; doi:10.1007/s00259-023-06326-w)
Supplement: Supplementary file 1 — Supplementary file1 (DOCX 606 kb) [file 259_2023_6326_MOESM1_ESM.docx]

**SUPPLEMENTAL FILES**

**Technical aspects of tumor segmentation in PSMA PET**

An effective separation of tumor lesions from the sites of normal uptake has been a challenge for many researchers dealing with the response assessment in PSMA PET. In this work, based on our own observation, a fixed SUV threshold of 4.0 was established as optimal for tumor segmentation. It ensured satisfactory separation of pathologic and physiologic sites in the majority of patients. In others, the overlapping structures of normal and pathologic uptake generated large confluent ROIs (region of interest), which sometimes required meticulous, time-consuming, manual refinement. Retroperitoneum is an example of such anatomic localization, where the semi-automatic system may at the same time delineate involved lymph nodes and intestinal uptake, which often additionally overlaps kidneys. On the other hand, adopting a higher global SUV threshold or using percental thresholding (e.g., 50% of the SUV_max_) may lead to substantial underestimation of the total lesion PSMA (TLP), especially in areas of high pathologic activity [1]. Gafita and co-workers proposed a complex method of adjusting variable SUV thresholds for the skeleton, lymph node, and visceral organ metastases, based on the liver background activity as a reference for physiologic PSMA uptake [2]. The authors proved its usefulness for tumor load estimation in highly-metastasized PCa patients.

Interestingly, we have noticed that if a patient presented only BMs without pelvic or abdominal LNMs, exclusion of physiological uptake in the liver, spleen, intestines and kidneys from the total volume of PSMA accumulating structures was easy to do with one “mouse click”. Due to the close proximity and overlapping of ROIs over these organs, they usually form one confluent cluster of ROIs.

**References:**

1. Seifert R, Herrmann K. Semiautomatically Quantified Tumor Volume Using 68Ga-PSMA-11 PET as a Biomarker for Survival in Patients with Advanced Prostate Cancer. J Nucl Med. 2020; https://doi.org/10.2967/jnumed.120.242057
2. [Gafita](https://pubmed.ncbi.nlm.nih.gov/?sort=date&term=Gafita+A&cauthor_id=30850484) A, [Bieth](https://pubmed.ncbi.nlm.nih.gov/?sort=date&term=Bieth+M&cauthor_id=30850484) M. qPSMA: Semiautomatic Software for Whole-Body Tumor Burden Assessment in Prostate Cancer Using 68Ga-PSMA11 PET/CT. J Nucl Med. 2019; <https://doi.org/10.2967/jnumed.118.224055>

**Figures and tables:**

Table 1S. Concordance between *early* **radiographic** (TLP) and *overall* **biochemical** (PSA) response in patients undergoing [^177^Lu]Lu-PSMA I&T RLT (n=40). TLP – total lesion PSMA (total tumor burden). PSA – prostate-specific antigen.

|  |  | Overall PSA |  |  |  |
| --- | --- | --- | --- | --- | --- |
|  |  | Complete response | Partial response | Stable disease | Progressive disease |
| Early | Complete response | 0 | 0 | 0 | 0 |
| TLP | Partial response | 0 | 10 | 1 | 1 |
|  | Stable disease | 0 | 9 | 7 | 3 |
|  | Progressive disease | 0 | 0 | 3 | 6 |
|  |  | κ = 0.3663 | | | |


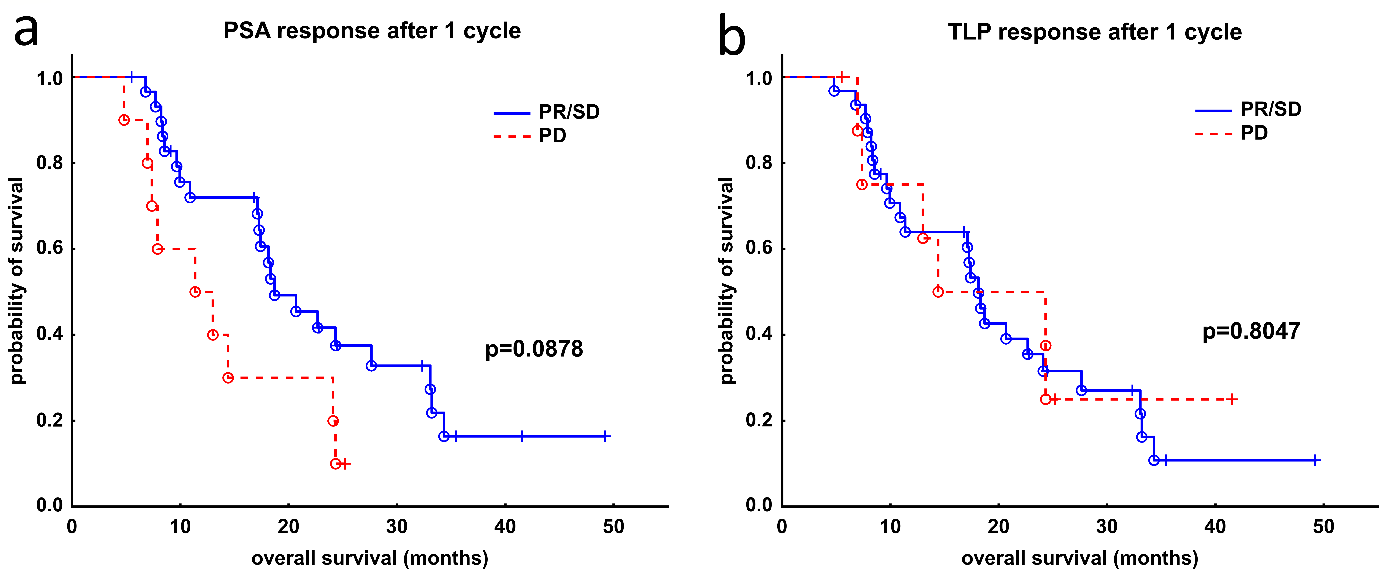


Fig. 1S. Kaplan-Meier analysis of survival stratified by the **early** biochemical (A) and radiographic (B) response (PR/SD vs. PD) based on the relative change of PSA and TLP, respectively, in 40 patients with mCRPC after one cycle of [^177^Lu]Lu-PSMA I&T. mCRPC – metastatic castration resistant prostate cancer.


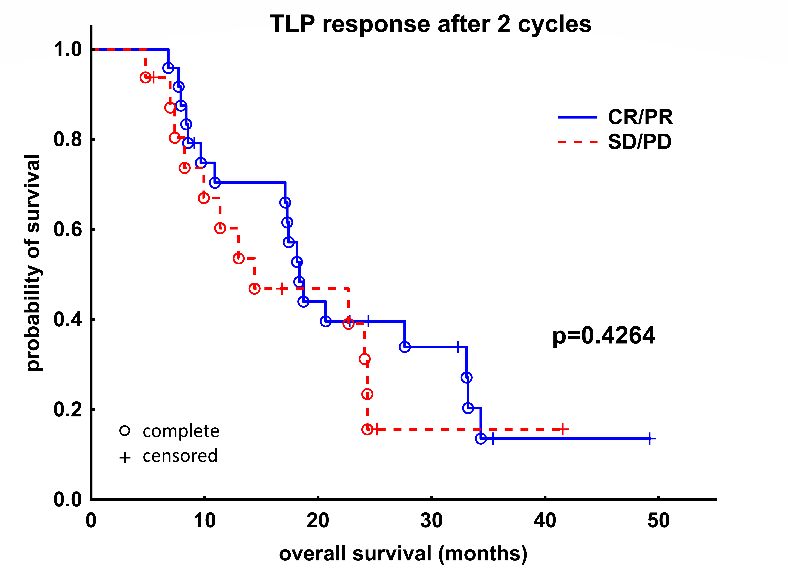


Fig. 2S. Kaplan-Meier analysis of survival stratified by the radiographic response (CR/PR vs. SD/PD) after 2 cycles of RLT based on the relative change of PSA in 40 patients with mCRPC after one cycle of [^177^Lu]Lu-PSMA I&T. mCRPC – metastatic castration resistant prostate cancer.

**
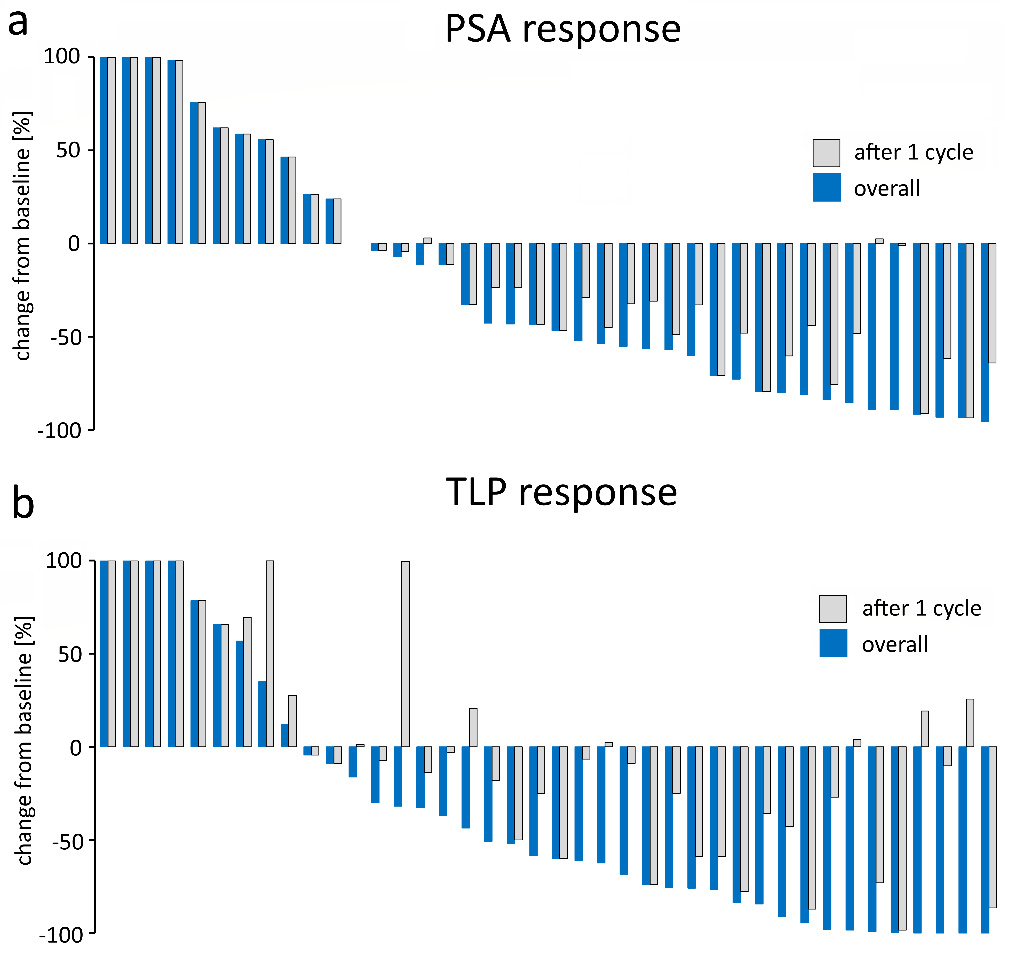
**

Fig. 3S. Waterfall plots of 40 patients showing relative percentage changes in PSA (A) and TLP (B) as a result of [^177^Lu]Lu-PSMA I&T radioligand therapy. Gray bars represent changes of a given parameter after one treatment cycle, while blue bars represent overall changes from baseline. Values over 100% were cropped for simplicity.


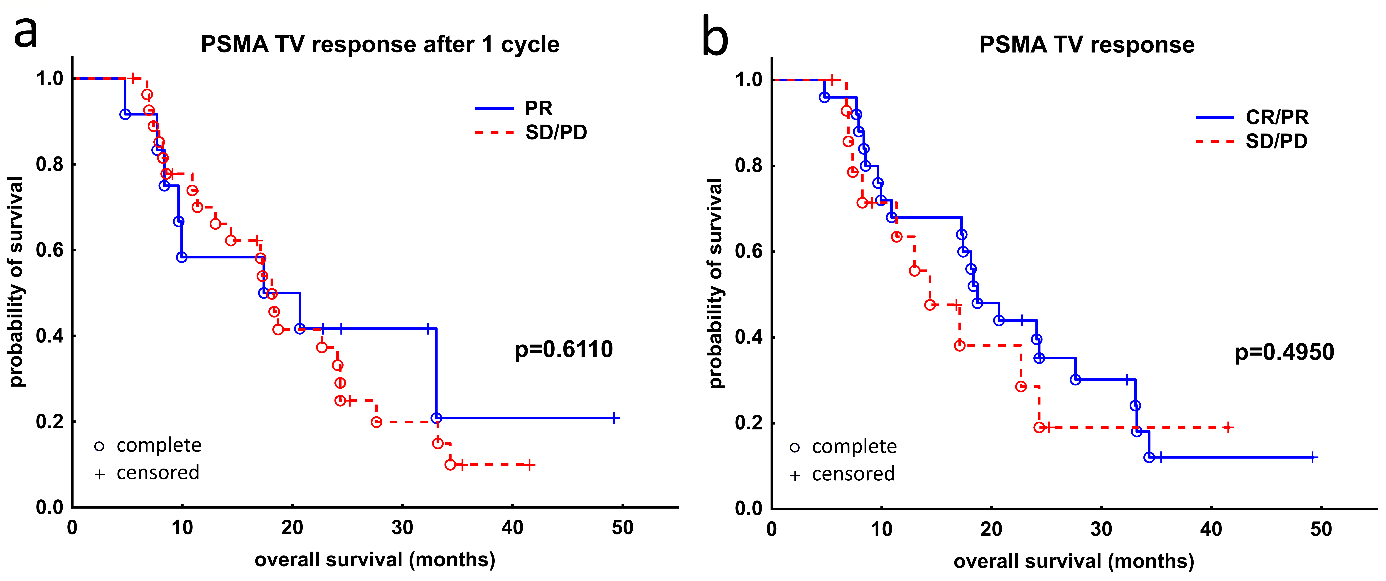


Fig. 4S. Kaplan-Meier analysis of survival stratified by the early (A) and overall (B) radiographic response ([CR]/PR vs. SD/PD) based on the relative change of PSMA-positive TV in 40 patients with mCRPC after [^177^Lu]Lu-PSMA I&T RLT. mCRPC – metastatic castration resistant prostate cancer. TV – tumor volume.


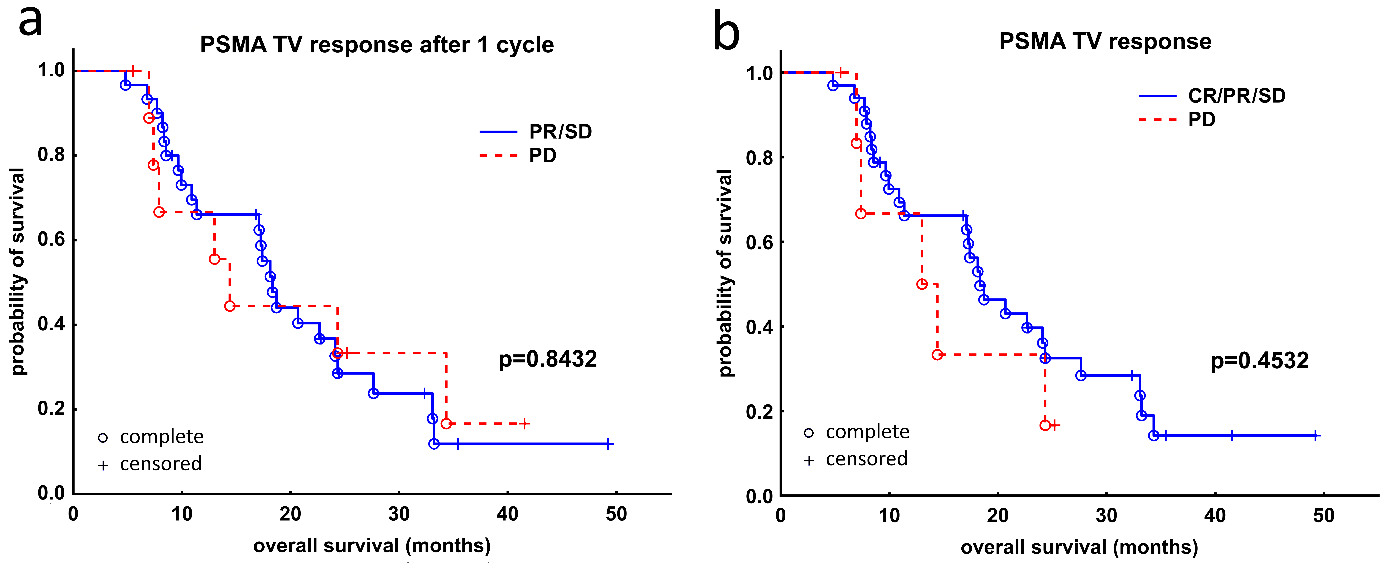


Fig. 5S. Kaplan-Meier analysis of survival stratified by the early (A) and overall (B) radiographic response ([CR]/PR/SD vs. PD) based on the relative change of PSMA-positive TV in 40 patients with mCRPC after [^177^Lu]Lu-PSMA I&T RLT. mCRPC – metastatic castration resistant prostate cancer. TV – tumor volume.
